# *“Sometimes I’ve gone home feeling that my voice hasn’t been heard”:* a focus group study exploring the views and experiences of health care assistants when caring for dying residents

**DOI:** 10.1186/s12904-016-0150-3

**Published:** 2016-08-19

**Authors:** Susan Fryer, Gary Bellamy, Tessa Morgan, Merryn Gott

**Affiliations:** 1 Manukau Locality, Counties Manukau Health, Manukau, New Zealand; 2 North West London Clinical Research Network, London, England; 3 School of Nursing, University of Auckland, Auckland, New Zealand

**Keywords:** Health care assistant, Aged residential care, Dying, End of life, Palliative, Older people, Care home, Nursing home, Residential aged care settings

## Abstract

**Background:**

In most developed countries, Health Care Assistants comprise a significant, and growing, proportion of the residential aged care workforce. Despite the fact that they provide the majority of direct care for residents, little is known about a key care aspect of their work, namely their experience of caring for dying residents.

**Methods:**

Twenty-six Health Care Assistants working in aged residential care facilities in Auckland, New Zealand participated in six focus group discussions. Focus groups were designed to explore the experiences of Health Care Assistants caring for imminently dying residents in aged care facilities and to identify barriers and facilitators to their work in this area. The focus groups were digitally recorded, transcribed verbatim and analysed using a general inductive approach.

**Results:**

Participants confirmed that Health Care Assistants provide the majority of hands on care to dying residents and believed they had a valuable role to play at this time due to their unique ‘familial’ relationship with residents and families. However, it was apparent that a number of barriers existed to them maximising their contribution to supporting dying residents, most notably the lack of value placed on their knowledge and experience by other members of the multidisciplinary team. Whilst a need for additional palliative and end of life care education was identified, a preference was identified for hands on education delivered by peers, rather than the didactic education they currently receive.

**Conclusion:**

Given ageing populations internationally coupled with a constrained health budget, the role of Health Care Assistants in most developed countries is likely to become even more significant in the short to medium term. This study makes a unique contribution to the international literature by identifying the barriers to caring for dying residents experienced by this valuable sector of the aged care workforce. These data have the potential to inform new, innovative, interventions to address the urgent need identified to improve palliative and end of life care management in aged care internationally.

## Background

Internationally, the number of deaths occurring in aged residential care (ARC) is increasing exponentially. Indeed, in many developed countries, including Canada, the United States of America, Australia, England and Wales and Switzerland, one half to one third of people >65 years now die in ARC [1–3]. ARC is for older people who can no longer live at home for reasons such as illness, disability, the needs of their carer, or because it is no longer possible to manage at home without help [4]. Unsurprisingly, then, as more people continue to live into advanced age throughout the developed world [5], these ARC facilities are increasingly becoming the place of death for a significant proportion of the population [2, 3, 6]. For example, in The Netherlands, over half of people (52.8 %) aged >80 years are currently dying in ARC settings and similar figures have been reported in New Zealand, with 47.9 % of older adults over 80 dying in ARC [7].

Given the growing number of people in advanced age dying in ARC, coupled with reports in many countries of sub-optimum end of life care [6, 8] there exists a significant need to improve palliative care provision within aged care settings. While research has identified a variety of barriers to optimum end of life care, such as a lack of palliative care competencies [9], an enduring focus on rehabilitation [10], and lack of time available for dying residents [11], the role that Health Care Assistants (HCAs) play in the provision of palliative care has been scarcely considered. This is particularly concerning for in line with many other developed countries, including the UK, US and Australia, ARC facilities in New Zealand are predominantly staffed by HCAs, who currently comprise 53 % of the workforce [12, 13] in contrast to Registered Nurses who make up 10 % of ARC staff. Such increased reliance on HCAs has been associated with chronic staff shortages and the need for cost-effectiveness [6, 14] Within New Zealand, HCAs are defined by the New Zealand Nurses Organisation as ‘an employee who is an auxiliary to the nursing team and is able to perform tasks in their position description relating to patient care and who works under direction of a registered nurse or midwife’ [15]. In practice, however, the role of the HCA remains markedly ambiguous [16–19]. Moreover, while it is known that HCAs are involved in the majority of direct care to residents [14] and have a critical role to play in the provision of chronic and end of life care [20], little research either within New Zealand or internationally has focused specifically upon their experiences in caring for dying residents [21, 22].

The limited evidence that is available indicates that HCAs experience considerable barriers to end of life care provision. For example, it has been reported that HCAs tend to avoid actively engaging in end of life discussions with dying residents or their families [23–25] with identified barriers to so doing including a lack of confidence, unclear role boundaries and inadequate communication skills training [9, 13, 26, 27]. Moreover, the absence of team work has also been identified as a barrier to good care [13].

However, given the significant role HCAs are playing in end of life care management, further research is obviously needed to ensure the contribution of this valuable, and growing, sector of the aged care workforce is optimised. It was within this context that the present study was designed to explore the experiences of Health Care Assistants in caring for imminently dying residents in aged care facilities and, in particular, to identify the barriers and facilitators to their work in this area. It has been recognised that palliative care research has been beset by definitional ambiguity and ‘end of life’ can be particularly difficult to define in relation to the oldest old given the likelihood of an unpredictable disease trajectory [28]. Therefore, for the purposes of this study, a decision was taken to focus on a very specific period, namely when residents are imminently dying. We define the term *dying* as when death is expected within hours to days [29].

## Methods

A qualitative approach was adopted to enable an in-depth exploration about an issue which is not well understood, namely HCA’s experiences of caring for dying residents. Focus groups, a research technique that collects data through group interaction on a topic determined by the researcher [30], were chosen as the method for data collection because they provide an effective way to explore participants’ perceptions and feelings about a given topic due to the negotiation and explanation inherent in the interactive group setting [30, 31]. We also considered the democratising process of focus groups, with their emphasis on giving people a platform for their voices to be heard, [32], pertinent to our study on HCAs whose opinions are overwhelmingly overlooked due to their ‘non-specialist’ status. For these reasons focus groups have become a common feature of nursing research in general [32], and more recently have been successfully employed in palliative care research to investigate optimal care delivery methods [9, 12, 13, 33].

HCAs were eligible to participate in the study if they met the following inclusion criteria: (1) currently working in one of the participating aged residential care facilities; (2) had experience of caring for a dying resident within the previous 12 months; and (3) fluently spoke  English. Participants were recruited from within one semi-rural region of Auckland, where there are seven residential aged care facilities located. They were recruited via poster advertisement. This method was chosen because Charge Nurse Managers were concerned about the potential for perceived coercion if they invited the participants directly.

**Fig. 1:**
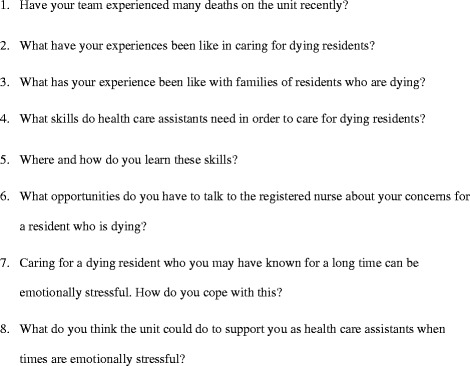
Focus group guide

All focus groups were facilitated by Susan Fryer (SF), who has a background as a palliative care nurse specialist, with the support of a Research Assistant, and took place at the participating facility in a six month period between 2011–2012. The focus group guide (Fig. 1) addressed the research aim and was informed by a critical review of the literature. The Research Assistant made field notes during the focus group and SF made extensive field notes following the focus group. Ethical approval was obtained from the University of Auckland Human Participants Ethics Committee (reference NTY/11/EXP/062) and all participants provided written informed consent to participate in the study.

### Data analysis

Focus group interviews were digitally recorded and transcribed verbatim. Two of the authors SF and Gary Bellamy (GB) independently read all the transcripts and discussed field notes. The first focus group transcript was independently coded by all three authors who met to discuss areas of commonality and divergence in coding; differences were resolved by consensus. This discussion provided the basis for the initial coding framework, which was subsequently refined during the process of analysis and then re-applied to all transcripts by SF. A decision was taken to manually code all data and not use a support package such as Nvivo to enable the lead author to remain close to the data. A general inductive approach to data analysis [34] was adopted because it enabled researchers to identify themes as they emerged from the raw data, which was especially important to this study [35] due to the lack of pre-existing frameworks focusing on capturing and analysing HCAs’ experiences.

Analyses were conducted in five stages: (1) Preparation of raw data files; (2) Close reading of the text; (3) Creation of categories; (4) Overlapping coding and uncoded text, and (5) Continuing revision and refinement of category system [34]. Methods advocated by Angen were applied to ensure rigour [36]. These included giving careful consideration and articulation to the research question, documenting evidence of the interpretive choices made by SF, identifying divergent cases, double coding, and working reflexively.

## Results

Of the seven facilities contacted only one care facility was not eligible for participation in the study as a death had not occurred there during the preceding 12 months: the remaining six facilities agreed to participate. Participating facilities reported, on average, 27 deaths in the preceding 12 months. Twenty- nine potential participants initially responded to the advertised poster; however, three subsequently withdraw for reasons including sickness and bereavement leaving a total of 26 participants, who took part in six one hour focus groups. All participants were female and the majority were aged between 40 and 59 years; 15 gave their ethnicity as NZ European, 4 identified as Māori, and the remaining 7 comprised a variety of nationalities including Tongan, Filipino and British (Table 1). The participants reported an average length of service of 8.1 years, with a range of 7 months to 26 years. Our following findings reflect a saturation point whereby all the themes arose in each focus group.

**Table 1:** Participants demographics (*n* = 26)

Ethnicity	Number of participants	Age	Number of participants
NZ European	15	<20	2
Maori	4	20–29	1
British	2	30–39	1
Tongan	1	40–49	8
South African	1	50–59	8
Filipino	1	60+	4
Fijian	1	No answer	2
Samoan	1		

Analyses revealed that HCAs positioned themselves as ‘part of the family’ and felt they played a valuable role in supporting dying residents and their families. However, it was apparent that their role in end of life care management was not optimised. Key barriers to ensuring high quality end of life care for residents were identified, including a lack of acknowledgement of the expertise and knowledge of HCAs by the wider multi-disciplinary team, who typically viewed them as ‘just a caregiver’. A need for further support to deal with both the emotional and practical aspects of this work was identified. Indeed, whilst participants reported that informal bereavement support was provided, it was apparent that this would benefit from being standardised and consistently provided. A preference for education delivered via peer support over didactic education sessions was identified. These themes are explored in more detail below.

### “Part of the family”

It was apparent that participants knew residents very well as a result of them being the key providers of hands on care. Indeed, many characterised their relationship as familial and acknowledged that this supported them in providing what they considered to be good quality care:

HCA1 “… *explain to them* [resident] *what you are doing, like we’re just going to wash your face, give your hands a wash, put a bit of lippy and perfume on. Treat them* [resident] *like they are part of the family. Think of how you would want your grandparents to be treated.”*

(Focus Group 2)

These relationships were reported to grow stronger over time and it was apparent that this supported the provision of individualised care, including when residents were dying. Indeed, prior knowledge of the individual was obviously key to HCAs providing care in a way they believed the resident would have wanted, particularly when residents were unable to communicate in their last few days. As the following participant explains:

HCA4 “…*you need to care for them* [a dying resident] *the way they expect, as if they could still speak to you. You usually know them well enough to know what they expect. I think if you can carry on doing that in their last days then it’s a good idea.”* (Focus Group 2)

Participants also reported spending a great deal of time with family members when their relative was dying and it was apparent that they played a key role in ensuring that the family’s practical and emotional needs were met at this time. For example, they discussed making them aware of tea making facilities and ensuring they had adequate pillows for their overnight stay should they wish to sleep at the facility. In addition, participants were conscious that many family members had never experienced the death of a close relative before and therefore identified a key role for HCAs in providing emotional support. As the following participant explains:

HCA1 “*We spend a lot of time with families and we put a lot of time in to them. They might not have dealt with a person dying before and it might be their first time. They don’t know what to do or how to act; often we are just supporting families through that dying phase as well”*

(Focus Group 1)

### “They think you’re ‘just a caregiver’”

The provision of day-to-day physical care to dying residents was viewed by participants as central to their role as HCAs. However, some participants expressed a degree of frustration that there was a lack of opportunity for them to share important information they held about a resident’s condition with either a doctor or the registered nurse. This led them feeling frustrated and questioning the value placed upon their role in the provision of end of life care by other members of the multi-disciplinary team. For example one participant said that:

HCA3 “Even though we’re caregivers, some of us have been through a lot in our lives not just with family members that have passed on, so, as an RN don’t write us off; if we come to them about a resident that we’re worried about; maybe listen.”

(Focus group 6)

Analyses identified that a key reason for HCA views not being listened to, particularly when residents were approaching death, was the professional hierarchy which existed within each of the facilities. As the following participant explains:

HCA2 “It (death) isn’t a new thing to me. I’ve got experience of my own family coming here, so I know what to expect. We’re not getting noticed for what [we] see.”

HCA3 “…*we* [HCAs] *are mature, not like we’ve no experience…that’s not acknowledged. Bit of a power thing I think.”*

(Focus Group 4)

Similarly another participant felt that:

HCA3 “Sometimes *I’ve gone home feeling that I’ve not been, um, my voice hasn’t been heard.”*

(Focus Group 1)

Indeed, participants recounted several stories of occasions when their concerns about dying residents had been largely ignored by more senior staff, typically the Registered Nurse on duty at the Facility. On most of these occasions, this refusal to listen to HCAs’ concerns resulted in significant negative repercussions. For example, the following excerpt highlights how the refusal of the trained nurse to take into account the HCAs’ knowledge and experience resulted in a poor outcome for the resident, the relatives and the staff:

HCA 2 *“We spend a lot of time with them* [residents]*; sometimes the RN’s just don’t listen. And you think, they are dying and you can just tell… just deteriorating*.

HCA 4 [Resident]*, she had pneumonia in the end I think. She was just getting weaker and weaker and not talking. I said to* [RN]*, you better call the family, but she didn’t. She* [resident] *died, and the family came in and got very upset with her because they expected to know. She should have called the family*.

HCA 5 *It was a bad mistake*

HCA 1 *You could tell with her breathing*

HCA 5 *We work with them* [residents] *all the time you know, and sort of know, we pick up things what’s wrong with them then go and tell them [RN’s] then it’s up to them, it’s not up to us*.

HCA 2 *They think, oh you’re just a caregiver; you don’t know what’s happening, but we’re with them every day.”*

(Focus Group 2)

### “It would be nice to get together and talk about it”

All participants reported a need to improve the emotional support provided by facilities to HCAs following the death of a resident. Indeed, having an opportunity to discuss the death of a resident appeared fundamental in helping HCAs deal with their repeated exposure to death and dying, as demonstrated by the following excerpt:

HCA 4 *“It would be nice to talk about it* [death]*, get together and talk occasionally*.

HCA1 *Not so much counselling but to be able to sit down and talk to somebody.”*

(Focus Group 1)

Participants reported that plans to implement a team debrief for emotionally challenging and difficult deaths had been discussed in the past in all participating facilities. However such debriefs had never actually been implemented and currently, the most common method of support for participants was informal peer support:

HCA 2 *“And we often can’t talk about it* [death and dying] *at home*.

HCA 3 *That’s why we do it at lunch*.

HCA 2 *We need to talk together* [on the unit] *because we understand each other so well.”*

(Focus Group 1)

Whilst these informal discussions were seen as valuable, the majority of participants were in favour of a more formal debriefing process, which acknowledged the impact that caring for dying residents and witnessing their deaths can exert on staff. It was evident that charge nurse should play a key leadership role in such a process, as their support was greater valued, even though it was currently offered in an unplanned and ad hoc way. As the following participant states:

HCA 3 “*I just have to say to [the charge nurse] “I need to talk”. She’ll say “just shut the door” and she’ll sit there with you.”*

(Focus Group 6)

Participants reported their own coping strategies when a resident died, identifying a number of ways in which they were able to ‘move on’. Being able to give the resident a dignified ‘goodbye’ by attending to the deceased body in a respectful manner was considered very important, but was difficult to achieve on occasions. Indeed, the following excerpt highlights the tensions that participants often experienced between managing a death appropriately and then having to welcome a new resident immediately:

HCA4 *“I don’t like it when someone has just passed* [died]*; the coldness of clearing the room, shifting their stuff while they’re still in the room. I’ve been upset so many times over that where they’ve [charge nurse] asked me to go clear a room and the undertaker is already there. It gets me every time crying*.

HCA2 *That’s cold to me*.

HCA1 *It’s a money making business*.

HCA2 *Give us time to get over it and then bring them* [new resident] *in the room*.

HCA1 *We’re told to clear the room at 3 pm and that someone new is coming in at 5 o’clock*.

HCA4 *There’s no time to spend getting it* [deceased belongings] *all together nice. What are the family going to think? I think it should be left a day*.

HCA3 *I don’t know how you girls feel, but I think what would be nice would be to get the room blessed for that soul that’s travelling on. It’s nice for that next person.”*

(Focus Group 6)

Despite the fact that participants viewed their role as working as part of the “facility family” the reality of working in a commercial business was evident in the turnover of residents they were witness to. Participants voiced their struggles with this quick turn-over, particularly in relation to managing their own bereavement experience.

### “I learn from the ones who have been here for a long time”

The majority of participants had a wealth of experience in caring for dying residents, with some having worked at the same facility for over 20 years. However, it was apparent that they did not generally discuss their knowledge and skills amongst themselves, or even recognise that they had developed expertise in this area. Interestingly, when given the opportunity to consider the sorts of skills they did have within the context of the focus group, there was a realisation amongst participants that they were skilled providers of end of life care, despite having no formal qualifications.

HCA 3 *“I think it’s something we just do, unless we talk about it [caring for dying residents] then it’s part of our everyday working life. I know that we have got skills that we don’t realise. Yeah, I suppose it is quite special.”*

(Focus Group 1)

When participants were asked to describe how they learnt to care for dying resident, most responded by stating they learnt their skills from working alongside other more experienced HCAs. Participants who were relatively new to their role and therefore identified specific knowledge and skills gaps were particularly in favour of this approach:

HCA 5 *“My first death was on a day shift; it was good because I had the help of other HCAs as well so I wasn’t forced into it. I went in with another HCA because I was obviously too scared to go in by myself but it was nice to see they weren’t suffering.”* (Focus Group 5)

As noted in the background, the majority of work to upskill HCAs within New Zealand, in line with many other developed countries, has been through the provision of didactic education programmes. Participants were therefore asked to consider whether this sort of formal education had a role in increasing HCAs’ knowledge of caring for dying residents. It was therefore interesting that many of the more experienced HCAs claimed that a classroom based approach would not be helpful to their learning, as they are used to being very practice focused:

HCA 2 “*Sometimes, not all the time, sometimes it [workshops] can be extremely boring*

[Everyone laughs]

HCA 1 *We’re so used to being on our feet all day that when you have to sit…*

HCA 2 *I could go to sleep*.

HCA 3 *It’s a different sort of pace.”*

(Focus Group 1)

## Discussion

This study addresses a significant gap in current knowledge in relation to understanding and exploring the views and experiences of HCAs caring for dying residents in aged residential care. Our findings confirm that HCAs play a critical role in the care of imminently dying residents in this setting, yet they experience significant barriers that limited their ability to maximise their contribution to end of life care delivery. Most notably, it was apparent that HCAs frequently felt that their unique skills and expertise were rarely acknowledged by the wider team, and as such they were also not adequately supported to manage their repeated exposure to death and dying.

It was apparent that key to their role in caring for dying residents was their strong relationship with residents and their families. The characterisation of this relationship as ‘familial’ resonates with the work of Phillips and Moss where staff also reported treating residents as ‘part of the family’ [9, 37]. Our findings also dovetail with recent work that shows that HCAs exert similar levels of emotional labour when providing palliative care in community-settings as we have found in the ARC context [17, 19]. The close resident/HCA relationship, which informed the HCAs’ in-depth knowledge of residents, has previously been acknowledged as central to the provision of holistic, individualised care by HCAs [13, 21, 38]. However, less attention has been paid to the way that this close relationship between HCAs and residents aids the provision of optimal end of life care [39]. Our findings indicate that because HCAs typically know residents well, and provide the majority of hands on care, they may often be first to notice that a resident is dying [13, 26]. Their potential contribution to palliative care management is therefore significant given previous research identifying a failure to recognise dying, and initiate a transition to a palliative approach to care, as a key barrier to ensuring high quality care at end of life [40]. It is therefore concerning that, when this type of information is relayed to a more senior colleague, our findings indicate that it is not always acted upon.

A tendency to ignore the views of HCAs because of a professional hierarchy which positions them ‘just as care workers’ has been reported elsewhere [9, 18]. For example, previous research indicates that suggests HCAs are not kept up to date with care plans for dying residents and left out of essential team briefings [12, 13, 41]. Similarly, within the acute hospital setting, a key barrier to a transition to a palliative approach to care has been identified to be the failure of senior medical clinicians to listen to the concerns of their more junior medical and nursing colleagues [40]. Within the aged care setting, it is apparent that there is an urgent need to consider ways of ensuring that the unique information HCAs hold about residents is able to inform their end of life care [42]. Butler-Williams et al. suggest that regular team building exercises coupled with the further development and evaluation of the support mechanisms available to HCAs would help improve cohesion between HCA and other ARC personnel by breaking down the workplace hierarchies [43], and in turn promoting positive [44] and less emotionally stressful [45] work environments.

Within a context where their views were not valued, it is unsurprising that participants were reluctant to acknowledge that they had developed expertise in end of life care management. However, it was apparent during the discussions that this was indeed the case and indeed, when given the opportunity to reflect, many participants did report feeling that they had amassed specific knowledge and skills in this area. Moreover, participants discussed how this knowledge and skills was shared amongst HCAs, with more experienced HCAs in particular taking a lead in mentoring and supporting new colleagues. That this type of ‘on the job’ peer learning was occurring is unsurprising given that HCAs work closely together the majority of the time [23, 38]. However, aside from Schell and Kayser-Jones [25], role modelling as a mode of education for HCAs has received little attention and palliative care education within ARC is typically provided through didactic education sessions, both internationally [46, 47], and in New Zealand [48]. Beyond this similarly, however, the majority of the HCA professional development literature remains disparate, with a lack of consistency surrounding the content of HCA training curricula and the expected duration of training programmes [49]. Given the urgent need to upskill HCAs in palliative care, particularly because their inadequate training has been identified as a major barrier to HCAs being able to provide optimal palliative care, the potential of utilising peer education to raise the standard of palliative care within this context warrants further research attention. As our results reveal, however, educational initiatives for HCAs must be coupled with wider organizational initiatives, fostering a more collaborative and integrative work environment, in order for the overall quality of palliative care to improve [6, 38, 50].

Another issue which also requires further attention is the need to build resilience amongst HCAs in managing their repeated exposure to death and dying. The experience of bereavement was particularly profound within the context of the familial relationships participants reported to have formed with residents, as discussed above. Indeed, within this context it was unsurprising that a need to develop systematic and consistent bereavement support was identified [27]. That there is currently little formal support provided by facilities to staff to help them with their experiences of bereavement is confirmed in the wider international research literature [24, 25] and requires urgent attention. It is known that unresolved loss can contribute to ARC staff experiencing burnout and job dissatisfaction [22, 51]. Given recent research showing that increased rates of burnout correlated with decreased willingness to engage in palliative care education among ARC staff, it follows that practitioners need to build resilience amongst HCA in order to ensure optimal end of life care [52].

### Strengths and limitations

This study has a number of strengths. The data were collected from six different residential aged care settings that served a large geographical area reduced the likelihood of local or site specific factors influencing the findings. Specific techniques to promote the robustness of the data collected were adopted. Our sample was also comparable to the general population of those working in ARC in New Zealand. All our participants were female which reflects the dominance of women in the care industry [45, 53] and in palliative care in particular [54]. We do acknowledge, however, that owing to the all-female cohort, our study cannot claim to speak for the experiences of male HCAs who an especially understudied group deserving of more attention in the literature. Our sample was also reflective of the ethnic variation of HCAs, in particular our study reflected the relatively high number of Māori and Pacific in this purportedly low-skilled and low paying job, which is typical of their marginalisation in the wider New Zealand economy [53].

Nevertheless, some limitations must also be acknowledged. Focus group dates were chosen by the charge nurse from each facility thereby limiting the number of participants able to take part in the study. Although a sufficient number took part in the study, this meant that the charge nurse was ultimately able to act as gate keepers, and thus our findings may not have encompassed views of the most dissatisfied. Participants were required to speak English which presents another limitation. This may have prevented some HCAs from participating who felt uncomfortable with their level of spoken English, but who had the relevant experience and knowledge. Given that emphasis on using immigrant labour to fill the gaps in the health care sector, in New Zealand [53] and internationally [55], future research is needed to investigate the culturally specific barriers and facilitators to involvement in end of life care in ARC.

## Conclusion

This study provides a unique insight into the experiences of caring for a dying resident from the perspective of HCAs and identifies key barriers to their involvement in end of life care which go beyond inadequate training and education, the focus of most development work to date. As such, the findings have significant practical implications given the growing numbers of people receiving end of life care within ARC settings internationally. In particular, they point to a need for HCAs to be acknowledged as expert members of the end of life care team and to ensure they receive sufficient support to fulfil this crucial role.

## References

[1] Reich O, Signorell A, Busato A (2013). Place of death and health care utilization for people in the last 6 months of life in Switzerland: a retrospective analysis using administrative data. BMC Health Serv Res.

[2] Broad JB, Gott M, Kim H, Boyd M, Chen H, Connolly MJ (2013). Where do people die? an international comparison of the percentage of deaths occurring in hospital and residential aged care settings in 45 populations, using published and available statistics. Int J Public Health.

[3] Cohen J, Bilsen J, Hooft P, Deboosere P, Wal G, Deliens L (2006). Dying at home or in an institution: using death certificates to explore the factors associated with place of death. Health Policy.

[4] DPS Guide. Terms and definitions. In*.* Australia DPS Guide; 2016

[5] Christensen K, Doblhammer G, Rau R, Vaupel JW. Ageing populations: the challenges ahead. The Lancet. 2009;374(9696):1196–1208. 10.1016/S0140-6736(09)61460-4 PMC2810516 19801098

[6] Froggatt KA (2001). Palliative care and nursing homes: where next?. Palliat Med.

[7] Cohen J, Gott M, Van Den Block L, Albers G, Onwuteaka-Philipsen B, Martins Pereira S, Pasman R, Deliens L (2015). Dying in place in old age: public health challenges. Palliative care for older people: A public health perspective.

[8] Reitinger E, Froggatt K, Brazil K, Heimerl K, Hockley J, Kunz R, Morbey H, Parker D, Husebo B, Reitinger E et al. Palliative Care in Long-term Care Settings for Older People: findings from an EAPC Taskforce. European Journal of Palliative Care. 2013;20(5):251-253.

[9] Phillips J, Davidson PM, Jackson D, Kristjanson L, Daly J, Curran J (2006). Residential aged care: the last frontier for palliative care. J Adv Nurs.

[10] Travis SS, Bernard M, Dixon S, McAuley WJ, Loving G, McClanahan L (2002). Obstacles to palliation and End-of-life care in a long-term care facility. Gerontologist.

[11] Wowchuk SM, McClement S, Bond J (2007). The challenge of providing palliative care in the nursing home. Int J Palliat Nurs.

[12] Phillips JL, Davidson PM, Jackson D, Kristjanson LJ (2008). Multi‐faceted palliative care intervention: aged care nurses’ and care assistants’ perceptions and experiences. J Adv Nurs.

[13] Hanson LC, Henderson M, Menon M (2002). As individual as death itself: a focus group study of terminal care in nursing homes. J Palliat Med.

[14] McKenna HP, Hasson F, Keeney S (2004). Patient safety and quality of care: the role of the health care assistant. J Nurs Manag.

[15] New Zealand Nurses Organisation and Listed Specified Hospices. Multi-Employer Collective Agreement. In*.* Wellington: New Zealand Nurses Organisation; 2012.

[16] Keeney S, Hasson F, McKenna H, Gillen P (2005). Nurses’, midwives’ and patients’ perceptions of trained health care assistants. J Adv Nurs.

[17] Lovatt M, Nanton V, Roberts J, Ingleton C, Noble B, Pitt E, Seers K, Munday D (2015). The provision of emotional labour by health care assistants caring for dying cancer patients in the community: a qualitative study into the experiences of health care assistants and bereaved family carers. Int J Nurs Stud.

[18] Spilsbury K, Meyer J (2004). Use, misuse and non-use of health care assistants: understanding the work of health care assistants in a hospital setting. J Nurs Manag.

[19] Herber OR, Johnston BM (2013). The role of healthcare support workers in providing palliative and end-of-life care in the community: a systematic literature review. Health Soc Care Community.

[20] World Health O (2002). Innovative care for chronic conditions: building blocks for action.

[21] McClement S, Wowchuk S, Klaasen K (2009). “Caring as if it were my family”: health care aides’ perspectives about expert care of the dying resident in a personal care home. Palliat Support Care.

[22] Latta L, Ross J (2010). Exploring the impact of palliative care education for care assistants employed in residential aged care facilities in Otago, New Zealand. J Soc Anthropol.

[23] Miskella C, Avis M (1998). Care of the dying person in the nursing home: exploring the care assistants’ contribution. Eur J Oncol Nurs.

[24] Beck I, Törnquist A, Broström L, Edberg A-K (2012). Having to focus on doing rather than being—Nurse assistants’ experience of palliative care in municipal residential care settings. Int J Nurs Stud.

[25] Schell ES, Kayser-Jones J (2007). “Getting into the skin”: Empathy and role taking in certified nursing assistants’ care of dying residents. Appl Nurs Res.

[26] Boockvar K, Brodie HD, Lacks M (2000). Nursing assistants detect behavior changes in nursing home residents that precede acute illness: development and validation of an illness warning instrument. J Am Geriatr Soc.

[27] McDonnell MM, McGuigan E, McElhinney J, McTeggart M, McClure D (2009). An analysis of the palliative care education needs of RGNs and HCAs in nursing homes in Ireland. Int J Palliat Nurs.

[28] Gardiner C, Cobb M, Gott M, Ingleton C (2011). Barriers to providing palliative care for older people in acute hospitals. Age Ageing.

[29] Stevenson J, Ellershaw J, Glare P, Christakis NA (2008). Prognostication in the imminently dying patient. Prognosis in Advanced Cancer.

[30] Morgan DL (1996). Focus Groups. Ann Rev Soc.

[31] Halcomb EJ, Gholizadeh L, DiGiacomo M, Phillips J, Davidson PM (2007). Literature review: considerations in undertaking focus group research with culturally and linguistically diverse groups. J Clin Nurs.

[32] Kevern J, Webb C (2001). Focus groups as a tool for critical social research in nurse education. Nurse Educ Today.

[33] Gott M, Seymour J, Ingleton C, Gardiner C, Bellamy G (2012). ‘That’s part of everybody’s job’: the perspectives of health care staff in England and New Zealand on the meaning and remit of palliative care. Palliat Med.

[34] Thomas DR (2006). A general inductive approach for analyzing qualitative evaluation data. Am J Eval.

[35] Elo S, Kyngäs H (2008). The qualitative content analysis process. J Adv Nurs.

[36] Angen MJ (2000). Evaluating interpretive inquiry: reviewing the validity debate and opening the dialogue. Qual Health Res.

[37] Moss MS, Moss SZ, Rubinstein RL, Black HK (2003). The metaphor of “family” in staff communication about dying and death. J Gerontol B Psychol Sci Soc Sci.

[38] Hirakawa Y, Kuzuya M, Uemura K (2009). Opinion survey of nursing or caring staff at long-term care facilities about end-of-life care provision and staff education. Arch Gerontol Geriatr.

[39] Nolan M, Featherson J, Nolan J (2003). Palliative care philosophy in care homes: lessons from New Zealand. J Adv Nurs.

[40] Gott M, Gardiner C, Small N, Payne S, Seamark D, Barnes S, Halpin D, Ruse C (2009). Barriers to advance care planning in chronic obstructive pulmonary disease. Palliat Med.

[41] Touhy T, Brown C, Smith C (2005). Spiritual caring: End of life in a nursing home. J Gerontol Nurs.

[42] Zheng NT, Temkin-Greener H (2010). End-of-life care in nursing homes: the importance of CNA staff communication. J Am Med Dir Assoc.

[43] Butler-Williams C, James J, Cox H, Hunt J (2010). The hidden contribution of the health care assistant: a survey-based exploration of support to their role in caring for the acutely ill patient in the general ward setting. J Nurs Manag.

[44] Pennington KMSRN, Scott JPRN, Magilvy KPRN (2003). The role of certified nursing assistants in nursing homes. J Nurs Adm.

[45] George E, Hale L, Angelo J. Valuing the health of the support worker in the aged care sector. *Ageing & Society* 2016, FirstView:1–19.

[46] Parks SM, Haines C, Foreman D, McKinstry E, Maxwell TL (2005). Evaluation of an educational program for long-term care nursing assistants. J Am Med Dir Assoc.

[47] Hanson LC, Reynolds KS, Henderson M, Pickard CG (2005). A quality improvement intervention to increase palliative care in nursing homes. J Palliat Med.

[48] Hospice New Z (2012). Fundamentals in Palliative Care.

[49] Keeney S, Hasson F, McKenna H (2005). Health care assistants: the views of managers of health care agencies on training and employment. J Nurs Manag.

[50] Finucane AM, Stevenson B, Moyes R, Oxenham D, Murray SA (2013). Improving end-of-life care in nursing homes: implementation and evaluation of an intervention to sustain quality of care. Palliat Med.

[51] Anderson KA (2008). Grief experiences of CNAs: relationships with burnout and turnover. J Gerontol Nurs.

[52] Frey R, Boyd M, Foster S, Robinson J, Gott M (2015). Burnout matters: the impact on residential aged care staffs’ willingness to undertake formal palliative care training. Prog Palliat Care.

[53] Badkar J (2009). The future demand for paid caregivers in a rapidly ageing society.

[54] Morgan T, Ann Williams L, Trussardi G, Gott M. Gender and family caregiving at the end-of-life in the context of old age: A systematic review. Palliative Medicine 2016. 10.1177/0269216315625857 26814213

[55] Hugo G (2007). Contextualising the’Crisis in aged Care’ in Australia : a demographic perspective. Aust J Soc Issues (Australian Council of Social Service).

